# An Abridged Theraphy

**Published:** 1898-12

**Authors:** 


### BOOK NOTICES.

An Abridged Therapy: Manual for the Biochemical Treatment of Disease. By Dr. Med. Schuessler, of Oldenburg. Twenty-fifth edition, in part rewritten. Translated by Professor Louis H. Tafel. Philadelphia: Bœricke & Tafel, 1898. Price, cloth, $1.00; by mail, $1.07.

Scarcely a book written for homœopathic physicians has had the immense circulation that this product of Dr. Schuessler's mind has enjoyed, it having passed through twenty-five editions! Hardly any book that has appeared for the use of the homœopathist has had as many editors as this one. Additions have been made not alone by the author, but by his various ambitious editors until the original has been swollen out to proportions so large that it can hardly be recognized as the modest little duodecimo we first saw thirty years ago, edited by Dr. Hering. The present edition is free, however, from any additions of others except a short biography of the venerable author, who died before he could see the finish of his last effort.

The before-mentioned popularity of this book is to our mind not far to seek, and does not depend upon such very superior clinical results over the ordinary method of finding a simillimum to the case to be treated.

It would seem that there are several factors which go to make up this popularity.

In the first place, it reduces the number of medicines that the practitioner need depend upon to twelve, instead of the vast array of remedies that go to make up the ordinary materia medica.

In the second place, it reduces the symptomatology enormously. There is no need, if we adopt this system, of having the swollen books of materia medica that burden our shelves and harass our minds in the daily task of healing the sick.

In the third place, it offers plausible theories of the action of medicines upon the human system, very welcome to most minds addicted to speculative attempts to account for the phenomena that occur in the sick.

In the fourth place, it affords a refuge for many, who, dissatisfied with the inefficient therapeutics of the old school of medicine, are not yet mentally trained for the acceptance of true Homœopathy, and want a middle ground to stand upon, that will exempt them from any obligation to practice Hahnemann's principle.

It may be summarized as a case of Homœopathy made easy; and that is what so many of us want. A very considerable proportion of the profession are seeking to reduce the practice of Homœopathy to a business routine like the ceaseless round of some machine; thereby saving time and labor and enabling them to increase their capacity for seeing patients in greater numbers.

This spirit of routinism with its advantages may be well illustrated by the development of the printing press. When Caxton, Franklin, and others practiced with their rude presses, where much effort only made one imprint at a time, and that on only one side of a sheet, their work was excessively slow. When the idea was elaborated of putting the type on a carriage and shoving it under a revolving cylinder on which were sheets of paper to receive the impressions, then was the rapidity of production enormously increased. When the idea was still futher elaborated and the type was built upon a cylinder and made to rotate against another rotating cylinder over which passed a long strip of paper, then was the highest possible speed of production attained, and as many printed papers turned out in one second as had been possible before with Franklin's presses in an hour.

In Homœopathy the spirit of progress(?) is along the lines that developed the printing press. The average professional mind seeks to reduce homœopathic practice to a routine business strongly suggestive of the idea of type built upon a rotating cylinder. It is not a method that will benefit the patient, though it is easier and more profitable to the practitioner.

Schuessler's bold idea is in perfect harmony with this spirit of routinism, and hence it has a greater impetus commercially than other books. But the reader need not think that as a system it improves our capacity to treat sickness. Whoever uses it will constantly find himself obliged to fall back upon symptomatology, and insensibly will be led outside the charmed circle of the twelve remedies, to others that the venerable author has either purposely excluded or simply ignored.

When any great discoverer comes forward with a perfectly new and unique idea the whole mass of humanity, finding it an innovation upon their usual and accepted modes of thought, at once reject it and refuse to consider any evidence in its favor. If the discoverer persist in forcing it upon them, they proceed to persecute him. If he withstand the persecution he will find a few individuals come out of the throng and accept the new teaching, and of course soon share with him in ridicule and persecution. But as the discoverer continues to maintain his doctrine, opposition to him more and more decreases until there comes a kind of toleration of his ideas. This toleration is succeeded in its turn by a sort of tacit assent in general and the persecution ceases. Those who thus give their assent are not really convinced of its truth, but are attracted to it by the prospect of some personal advantage or profit. Consequently they advocate it by speech and distinguish themselves by their partisanship while in practice actually nullifying it or ignoring it.

If a pretender arise who offers a mutilated form of it as a substitute for the original doctrine, these partisans will flock to him and become his disciples, leaving the original discoverer with but the smallest possible following.

Now the sketch here drawn fits the history of Hahnemann and his doctrine. It is, indeed, an epitome of it. From total rejection of it Hahnemann passed to the stage of acceptance by a few faithful followers, then to its seeming acceptance by a large and unthinking minority, and then to the stage where pretenders attempted to teach modifications. Thus we find followers who wrote rival organons, or assumed to improve his writings by perverted translations, or sought to graft upon his plain announcement of a law of cure, the cumbrous speculative philosophy of the old school, apparently with a view of affording a refuge for those who choose to profess the principle, but not being convinced of it, do not intend to practice it.

This, we think, is the attitude of Schuessler toward Hahnemann. He simply affords a refuge to those who *profess* Hahnemann's principles, but who are not convinced of them and will not practice them. As these are the great majority of Hahnemann's so-called disciples, the great popularity of Schuessler's work is in part explained.

The work before us now in its completest form strikes us as being in the very highest degree speculative. It is an attempt to give rational explanations upon material lines of the action of delicate potentized remedies made, of course, on the plans of Hahnemann, but nevertheless prescribed, not upon the basis of the law of similars, but upon this fanciful theory of chemical reactions.

Take for example his views upon Natrum-muriaticum or table salt. He says at page 58 that table salt entering the human system as a combination between soda and chlorine, meets soda in the cells that is not combined with chlorine. The chlorine immediately leaves the soda with which it was combined when it entered the cells, and joins with that other soda which it finds already established there, and thus forms Natrum-muriaticum over again.

Thus the chlorine is invested by our venerable author with a selective power which approaches mentality. Chemistry teaches that where two substances of different chemical characteristics are found united, it is because of chemical affinity. That this combination is in whole or in part a satisfaction of this affinity, and the combination will remain quiescent until some other agent with higher affinity comes in contact with it when the first combination is broken, one of the constituents is liberated, and a new combination is formed.

But Dr. Schuessler teaches that chlorine combined with a metal in a certain compound has a higher affinity for some other portion of that same metal with which it happens to meet than that with which it already has united, and therefore will leave its first combination to unite with this second, although both are the same metal. That soda in combination has a less affinity for the element with which it is united than another portion of soda with which that element happens to meet. This is an absurdity and is actually laughable. It shows a want of clear understanding of the phenomena of chemistry.

In this same connection Dr. Schuessler teaches that an attenuated dose or potency of salt can cause endosmose.

This is in direct opposition to the meaning of endosmose, which is that if two bodies of water, the first one denser than the second by reason of its having in solution some salt—say table salt—are separated by a wall made of an animal membrane, the first or denser solution will pass through the membrane to increase the density of the other or second body of water, while the second will pass through toward the denser fluid to dilute it. This exchange will go on until both bodies of fluid are of the same density, when it will cease. Hence it is a phenomenon dependent upon the presence of ponderable quantities. Yet Dr. Schuessler seems to think that the presence of table salt in infinitesimal quantities is quite competent to produce the same result.

Errors like these invalidate the whole book (for of course there are others less glaring) and destroy its scientific value. Meanwhile former editors of Schuessler's book have acknowledged right in the preface that these twelve tissue remedies could not be well used without provings in the regular Hahnemannian way. (See review of Bœricke & Dewey's edition of Schuessler in THE HOMŒOPATHIC PHYSICIAN for May, 1893, page 300.)

This journal has never been able to unreservedly commend Schuessler's system. We have spoken moderately in disapproval of every edition of it which has ever been brought to our notice, except perhaps that of Bœricke & Dewey before mentioned.

Dr. Lippe, Dr. Fellger, Dr. Wells, of Brooklyn; Dr. Henry N. Guernsey, and others who were distinguished by their success in the practice of pure Homœopathy and their bold and unwavering endorsement of it, strongly disapproved of the Biochemic theory.

Dr. Lippe loudly execrated what he called "Schuesslerism," and classed its author among the enemies of Homœopathy. He did not hesitate to accuse Schuessler of having invented Biochemism as a means of preventing the progress of the homœopathic principle by affording a refuge, as before explained, for those minds who wished to display an outward adherence to Homœopathy while carrying on a secret opposition to it.

Dr. Fellger complained of the editorial opinions of this journal concerning Biochemism as not being severe enough, and Dr. Wells would not condescend to discuss it.

Certain it is that it exhibits a want of profound knowledge of chemistry and an assumption of reactions which are not confirmed by experimental research.

It appears to have been constructed theoretically in the doctor's study with the aid of a few books of reference, one or two of which are decidedly speculative in their views and by a knowledge of chemistry which is certainly superficial and to our mind totally insufficient for the purposes sought to be attained. Thus the whole system of Biochemistry belongs to the domain of speculative medicine, and that is just what Homœopathy seeks to avoid.

